# Intraspecific competition and individual behaviour but not urbanization affect the dietary patterns of a generalist avian predator

**DOI:** 10.1038/s41598-023-37026-y

**Published:** 2023-06-24

**Authors:** Pedro Romero-Vidal, Álvaro Luna, Lola Fernández-Gómez, Joan Navarro, Antonio Palma, José L. Tella, Martina Carrete

**Affiliations:** 1 Department of Physical, Chemical and Natural Systems, Universidad Pablo de Olavide, Seville, Spain; 2 Department of Conservation Biology, Estación Biológica de Doñana (CSIC), Seville, Spain; 3 Department of Health Sciences, Faculty of Biomedical and Health Sciences, Universidad Europea de Madrid, Madrid, Spain; 4 Department of Applied Biology, Centro de Investigación e Innovación Alimentaria (CIAGRO-UMH), Universidad Miguel Hernández, Elche, Spain; 5 Institut de Ciències del Mar (ICM-CSIC), Barcelona, Spain

**Keywords:** Ecology, Zoology

## Abstract

Urbanization has reshaped ecosystems and changed natural processes, driving an intense transformation of biomes, biotic community composition and diversity. Despite the growing interest in studying urban ecology over the last decades, the consequences of these changes on species occupying these ecosystems are not yet fully understood. Trophic generalism and tolerance to human disturbance have been proposed as two key traits in the colonization of urban environments. However, most studies focused on species’ average traits, paying less attention to the potential role of inter-individual variability. Here, we examined diet specialization in urban and rural breeding pairs, as well as its relationship with individual behaviour and intraspecific competition, using the burrowing owl as a study model. Our results show that both urban and rural breeding pairs behaved as trophic specialists. The diet of burrowing owl breeding pairs followed a gradient from coleopteran- to micromammal-dominated, which is related to individual behaviour (bolder individuals consuming more coleopterans than shyer ones). Besides, pairs distant from others showed a more diverse diet than those experiencing higher levels of intraspecific competition. Models fitted separately for each habitat showed that the proportion of micromammals in the diet of urban breeding pairs was related to their behavior, while the diet of rural pairs was not affected by individual behavior but by intraspecific competition. However, despite the strong selection of tame and more explorative individuals in urban environments and the higher density they reach in this habitat type, they did not differ in their degree of diet specialization from rural conspecifics. Although it would be necessary to evaluate prey availability on a small scale, our results suggest that burrowing owl breeding pairs behave as specialists, despite the generalist character of the species, and that this specialization is not affected by the occupation of urban environments but to individual behaviour and intraspecific competition.

## Introduction

Urbanization is one of the main drivers of global change^1–3^, and biodiversity loss (the so-called ‘biotic homogenization process’^4–6^). However, species response to urbanization varies, and there are many examples of animals adapting and even flourishing in these habitats^7–10^. Species that can colonize urban habitats mostly show a high degree of environmental tolerance, for example in terms of habitat breadth and geographic range size^6,11^. Although the mechanisms behind this pattern remain poorly studied, behavioural, physiological, and ecological flexibility have been proposed to explain the ability of organisms to tolerate disturbed habitats. The underlying assumption is that more tolerant, generalist species are composed of individuals capable of exploiting the full range of available resources, even adaptively shifting their niche when necessary, which allow them to cope with highly modified habitats, such as urban environments^12,13^.

In recent years, there have been a growing number of studies showing that generalist species are actually composed of individuals whose diets represent a spectrum of specialization within the overall feeding resource of a population^13–16^. This recognition about differences among individuals in ecological niche exploitation^14,17^ implies that within a species (or population), individuals can segregate by habitat and/or diet composition^18–20^ and be subject to different selective forces that can promote changes in the frequency of phenotypes, leading to population divergence^21^. In line with these results, Carrete and Tella^22^ have shown that the ability of birds to colonize urban habitats is related to their intraspecific (interindividual) variability in fear of humans. Although habituation has traditionally been considered the most likely explanation for differences in fear to humans across urbanization gradients^23–25^, these same authors have demonstrated the high repeatability of this behaviour across an individual’s adulthood^9,26^ as well as its heritability^27,28^, leaving a small margin for behavioural flexibility^29^. Thus, the process of urban colonization appears to be the result of tame individuals within a species with high interindividual variability in their fear of humans crossing the disturbance boundary^26,30^. This approach, which recognizes that individuals within a species (or population) are not ecological equivalents, bears similarities to the concept of ecological niche specialization, suggesting that individuals with particular behavioural profiles may also exhibit different degrees of niche specialization due to differences in their foraging behaviour^31^. Moreover, predation release improves the demographic parameters of urban individuals, increasing their breeding density compared to their rural conspecifics^32,33^. Several correlational studies have shown a positive relationship between population density (as a measure of intraspecific competition) and the degree of individual specialization^34–36^, while others pointed out that intraspecific competition determines behavioural diversification in microhabitat use^37^ or the inclusion of novel resources through increased interindividual variation^38–40^, thus reducing individual specialization. However, individual phenotypic variability (in terms of behavioural and foraging patterns) has been studied separately^31^, and more rarely considering differences in intraspecific competition^41^.

Here, we examined the trophic niche of urban and rural burrowing owls (*Athene cunicularia*), a predator species widely used as a model for studying avian urban colonization. Previous work has shown that the colonization of urban environments by burrowing owls has involved the selection of bolder individuals^42,43^. Moreover, predation release has allowed urban populations to reach higher demographic parameters than rural ones, which has favoured an increment of their breeding densities^33,44^ and, combined with behavioural changes, has reduced natal and breeding dispersal propensity, inducing small-scale genetic differences between urban and rural populations^43,45^. Some studies have proposed that the generalist diet of burrowing owls has also allowed them to cope with the challenges posed by new urban food sources^46–48^. However, despite the extensive literature comparing diets of urban and rural populations of different avian species^47,49–51^, there are no individual-based approaches that assess whether strong behavioural and density changes associated with urban colonization may be related to differences in the trophic niche of urban and rural birds.

## Results

We analyzed a total of 78 food samples (including pellets and prey remains) collected from 40 different burrowing owl pairs (2 pairs were sampled in a single year), 17 located in rural and 23 in urban areas. In total, we identified 5,890 different prey items from a variety of vertebrate and invertebrate taxa (Table S1). Arthropods constituted the most important prey group in number of individuals (mean number of individuals/sample = 78.64, SE = 75.07), mainly represented by coleopterans (81.44% of all arthropods). Vertebrates, which were less abundant (mean number of individuals/sample = 8.87, SE = 6.63), were mainly micromammals (81.30% of vertebrate prey). Prey biomass ranged from 0.01 g (*Camponotus* spp., Order Hymenoptera, Family Formicidae) to 400 g (*Rattus norvegicus*, Order Rodentia), with mammals representing the largest proportion of the biomass consumed by owls (80.37%).

**Figure 1:**
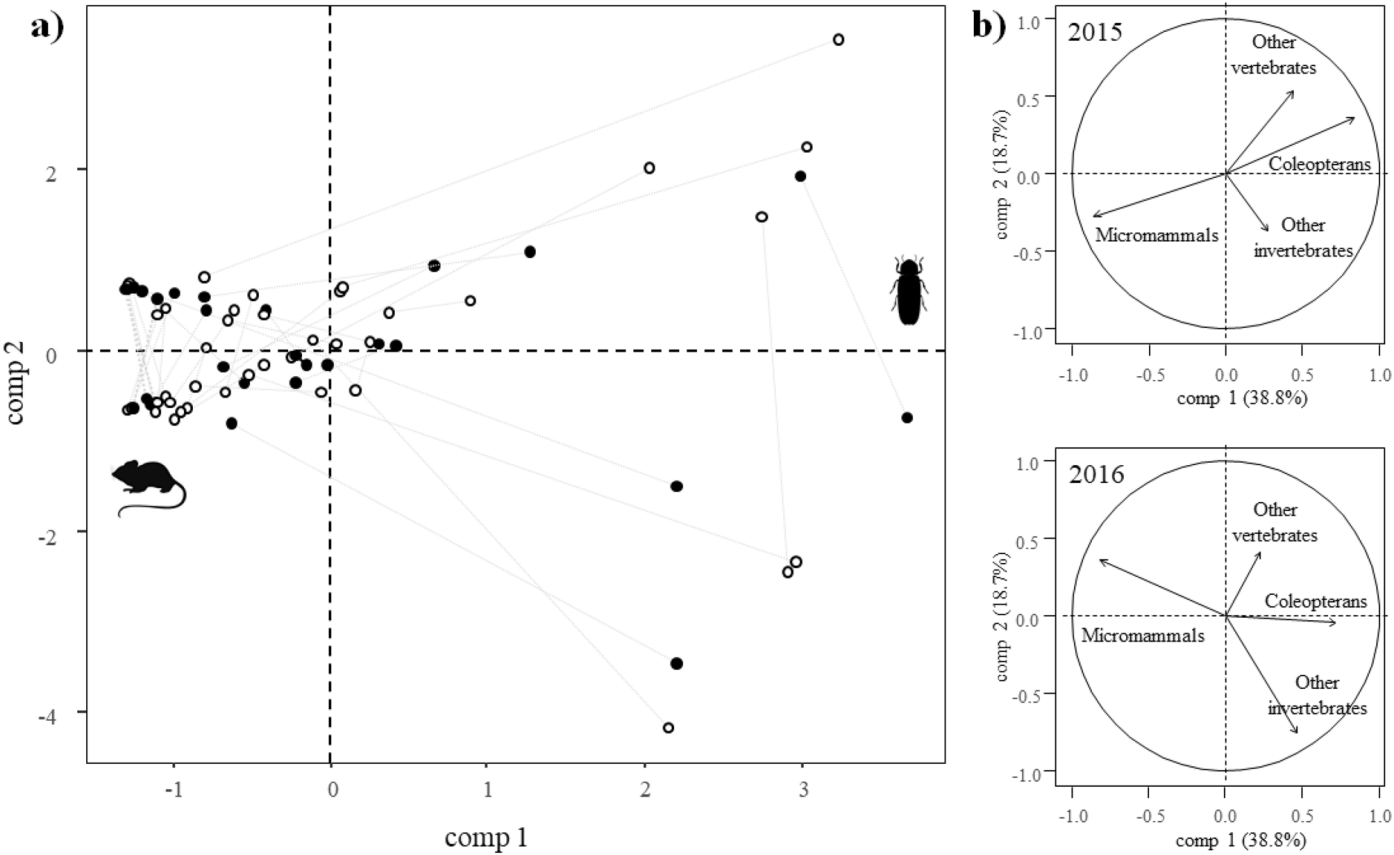
(**a)** Multiple Factor Analysis (MFA) to assess the dietary pattern of breeding pairs of urban and rural burrowing owls (white and black dots, respectively). Grey lines connect breeding pairs in the two years sampled, length being proportional to the divergence in the dietary pattern of each one. (**b)** Variable contributions to the MFA in 2015 and 2016.

**Figure 2:**
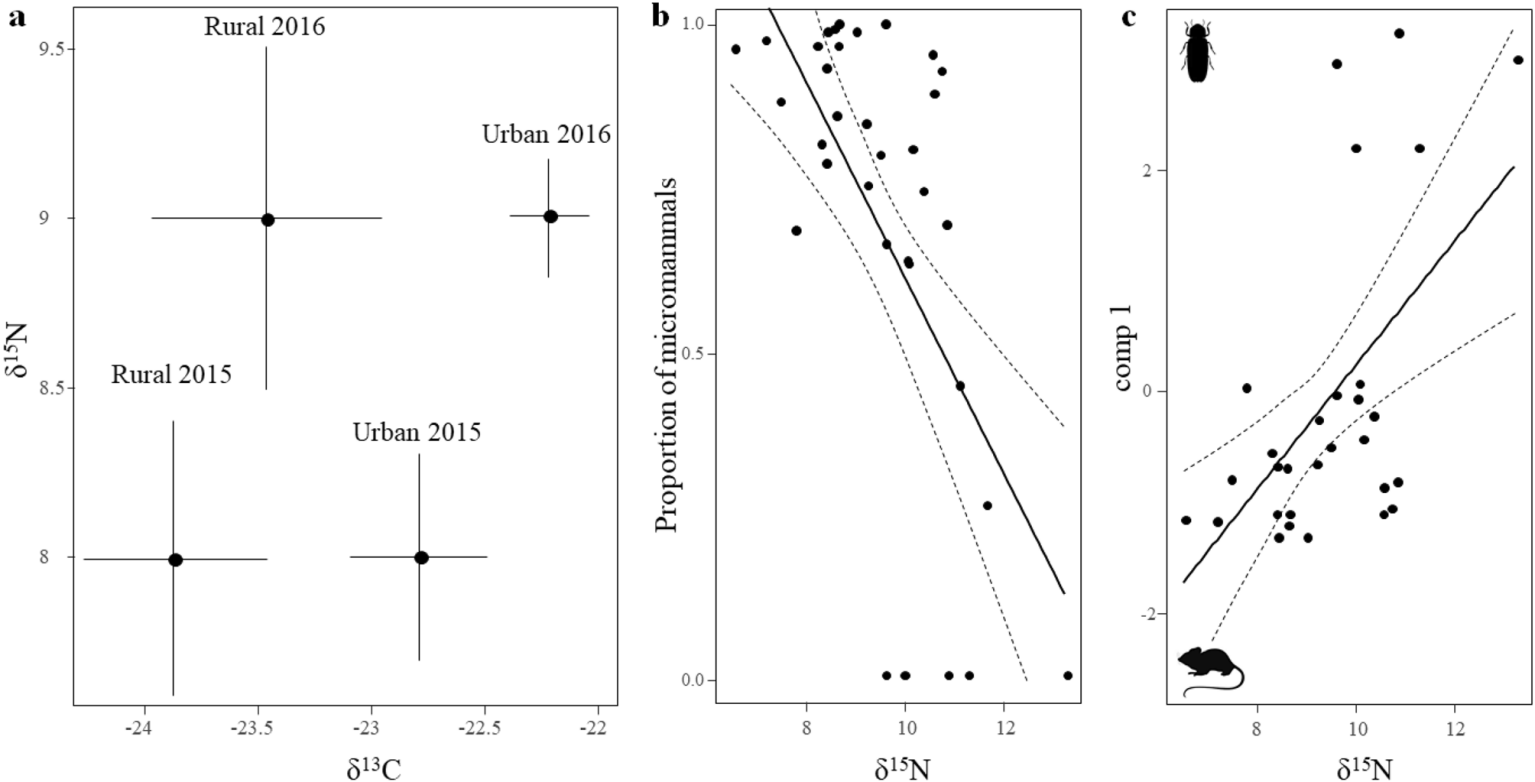
(**a**) Isotopic traces of δ^13^C and δ^15^N (mean ± SE) in the blood of burrowing owls living in urban and rural areas in the two study years and their relationship with the dietary pattern of the breeding pairs, estimated as (**b**) the proportion of micromammals and (**c**) the first component (comp 1) of a Multiple Factor Analysis (MFA; positive values can be interpreted as a descriptor of invertebrate-dominated diets, whereas negative values are indicative of breeding pairs preying primarily on micromammals). International standards for stable isotopes are reported vs. reference standards (see “Materials and methods” for details).

The MFA analysis conducted to describe the dietary patterns of the breeding pairs in 2015 and 2016 yield one dimension with an eigenvalue > 1 (38.83% variance explained; Table S2). This first dimension (comp 1) was negatively correlated with the proportion of micromammals in the diet, and positively correlated with the proportion of coleopterans (Table S2). Thus, positive values can be interpreted as a descriptor of invertebrate-dominated diets, whereas negative values are indicative of breeding pairs preying primarily on micromammals (Fig. 1).δ^15^N values for the burrowing owl breeding pairs did not differ between years (*p* > 0.18) and habitats (*p* > 0.37; Fig. 2a), but were related to comp 1 (estimate: 0.54, SE = 0.26, χ^2^ = 4.40, *df* = 1, p = 0.0358) and, thus, the proportion of micromammals in the diet (estimate: − 0.70, SE = 0.21, χ^2^ = 10.73, *df* = 1, *p* = 0.0011; Fig. 2b). δ^13^C values, however, differed between years (χ^2^ = 31.46, *df* = 1, *p* < 0.0001), and habitats (estimate for urban: 1.08, SE = 0.41, χ^2^ = 6.98, *df* = 1, *p* = 0.0083; Fig. 2a), and were not related to comp 1 (χ^2^ = 1.04, *df* = 1, *p* = 0.3074) nor to the proportion of micromammals in the diet (χ^2^ = 0.68, *df* = 1, *p* = 0.4091; Fig. 2c). These results support that the diet of burrowing owl breeding pairs followed a gradient from coleopteran- to micromammal-dominated (significant relationship between δ^15^N and comp 1 and the proportion of micromammals in the diet), and that urban and rural individuals obtain their feeding resources in the same habitat where their nests are located (differences in δ^13^C between urban and rural pairs).

The Total Niche Width (TNW) and Within Pair Component (WPC) obtained for urban and rural burrowing owls were similar between years (TNW: 2015: urban breeding pairs: 2.35, rural breeding pairs: 2.41; 2016: urban breeding pairs: 2.60, rural breeding pairs: 2.86; WPC: 2015: urban breeding pairs: 1.69, rural breeding pairs: 1.78; 2016: urban breeding pairs: 1.82, rural breeding pairs: 2.03). Thus, the value of the WPC/TNW ratio was also similar between habitats and years (2015: urban breeding pairs: 0.72, rural breeding pairs: 0.74; 2016: urban breeding pairs: 0.70, rural breeding pairs: 0.71). Monte Carlo simulations used to test the null hypothesis that all breeding pairs sampled equally from the population diet distribution show that urban and rural burrowing owls behave as specialists in both 2015 and 2016 (Monte Carlo resampling; all *p* = 0.001). Accordingly, IS values (and associated Monte Carlo simulations) also pointed towards specialization of urban and rural breeding pairs, with similar values for the entire study period (IS: 2015: urban breeding pairs: 0.51, rural breeding pairs: 0.51; 2016: urban breeding pairs: 0.70, rural breeding pairs: 0.71, all *p* < 0.001).

The proportion of the breeding pairs’ diet dominated by micromammals (or Coleopterans; Table S3) as well as their degree of overlap with respect to the diet of the whole population (Psi), remained similar in the two consecutive years (Repeatability, Table 1). In contrast, parameters such as prey richness and diversity or consumption of non-coleopteran invertebrates or non-mammal vertebrates changed over the study period (Table 1 and S3). Thus, although breeding pairs may change their diet composition between years, they do not exploit the full range of resources consumed by the population as a whole but tend to use a specific part of the full spectrum (i.e., mainly coleopterans or micromammals).

**Table 1:** The first five models obtained to assess differences in the diet parameters between urban and rural burrowing owl breeding pairs. Models included habitat (urban/rural), individual behavior (measured as flight initiation distance, FID), and intraspecific competition (measured as the distance to the nearest breeding pair, nnd, and the relative position of each breeding pair within the spatial distribution of the entire population, aggregation) as explanatory variables, year as a fixed factor and the identity of the breeding pair as a random term.

Diet parameter	Repeatability	Model selection					Model averaging			
		Models	*df*	AICc	ΔAICc	w	Variable	Estimate	2.5%	97.5%
Richness	0.21 (0, 0.51)	Nnd	4	417.69	0.00	0.11	Nnd	1.01	− 0.23	2.26
		Aggregation	4	417.98	0.29	0.09	Aggregation	− 1.03	− 2.27	0.21
		Aggregation + Year	5	418.09	0.39	0.09	Year (2016)	− 1.41	− 3.59	0.77
		Null	3	418.47	0.78	0.07	Habitat (urban)	− 1.54	− 4.31	1.23
		Habitat	4	418.89	1.20	0.06	FID	0.47	− 0.85	1.79
Diversity	0.01 (0, 0.35)	Nnd	4	104.59	0.00	0.30	Nnd**	0.13	0.01	0.25
		FID + Nnd	5	106.05	1.46	0.14	FID	0.06	− 0.06	0.18
		Nnd + Habitat	5	106.62	2.02	0.11				
		Aggregation	4	106.67	2.08	0.11				
		Null	3	107.43	2.84	0.07				
Micromammals	0.31 (0, 0.57)	Null	3	45.75	0.00	0.15	FID*	0.06	0.00	0.15
		FID	4	46.27	0.52	0.11	Aggregation	− 0.05	− 0.13	0.03
		Aggregation	4	46.58	0.83	0.10	Nnd	0.04	− 0.04	0.12
		Nnd	4	46.90	1.14	0.08	Year (2016)	− 0.04	− 0.17	0.10
		Year	4	47.70	1.95	0.06				
Psi	0.34 (0.01, 0.62)	Null	3	− 85.30	0.00	0.21	Year (2016)	− 0.02	− 0.07	0.03
		Year	4	− 83.88	1.41	0.10	nnd	− 0.01	− 0.04	0.02
		Nnd	4	− 83.61	1.68	0.09	Urban	0.02	− 0.05	0.09
		Habitat	4	− 83.38	1.92	0.08				
		FID	4	− 83.15	2.15	0.07				

The repeatability of each parameter (and its 95% confidence interval) was calculated using the null model (i.e., models without including explanatory variables). Richness: number of prey species in the diet; Diversity: diversity of prey species (Shannon index); Micromammals: proportion of micromammals in the diet; Psi: proportional similarity index used to describe the overlap between the diet of a pair and the diet of the entire population (PSi approaches 1 when pairs consume prey in direct proportion to the entire population, decreasing toward 0 in case of specialization). k: number of parameters, AICc: Akaike Information Criterion corrected for small sample sizes, ΔAICc: the difference between the AICc of model *i* and that of the best model (i.e. the model with the lowest AICc), w: Akaike weights. Estimates and 95% confidence intervals (2.5% and 97.5%) were assessed after model averaging. **: variables receiving strong support (i.e., the 95% confidence interval did not overlap with zero), *: variables receiving weak support (i.e., the 95% confidence interval barely overlap with zero).

**Figure 3:**
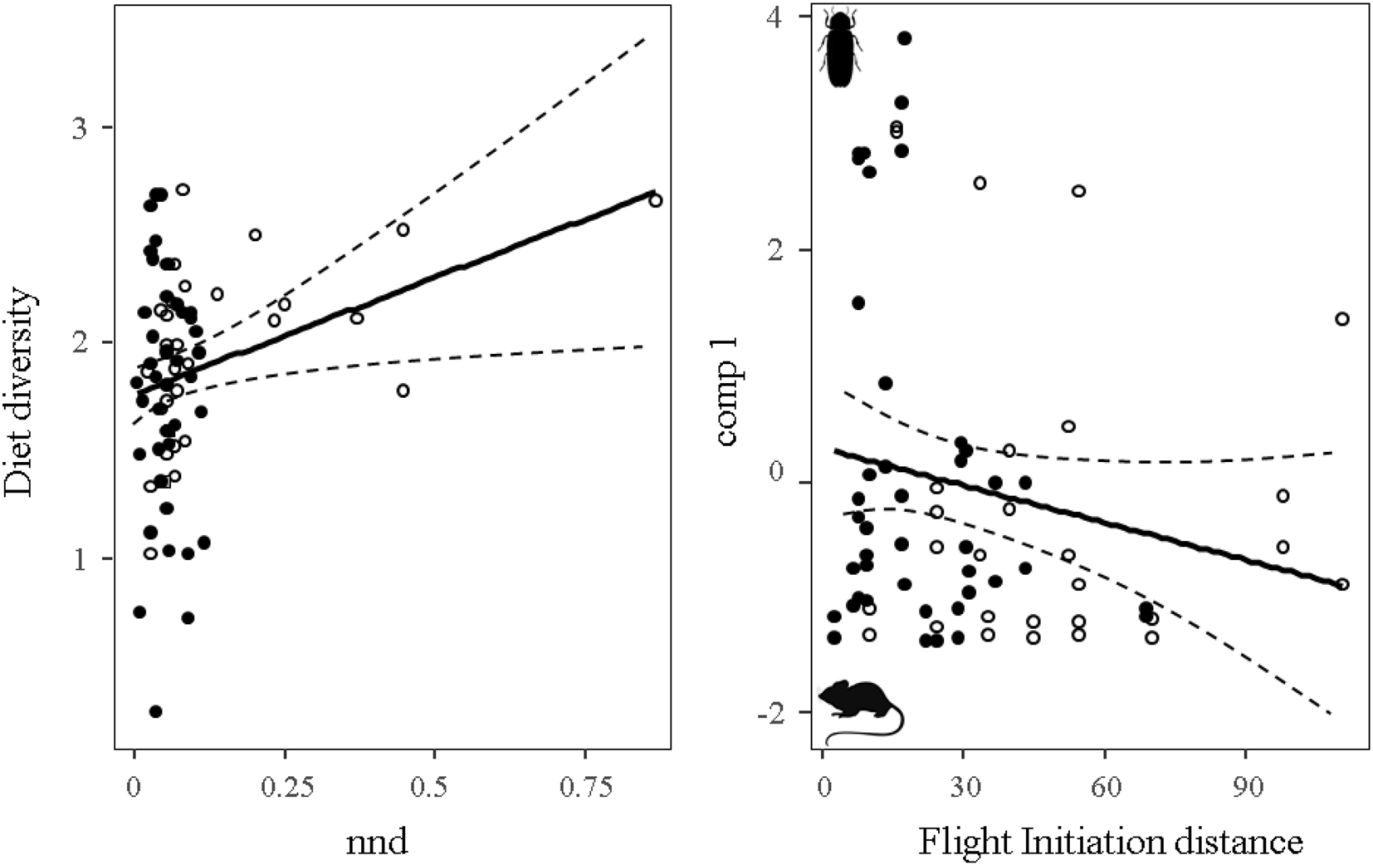
Relationship between (**a**) intraspecific competition (measured as the distance to the nearest neighbour breeding pair, nnd) and (**b**) individual behaviour (measured as the flight initiation distance of breeders, FID, in m) of urban (black dots) and rural (white dots) breeding burrowing owl pairs and their dietary patterns (diversity and comp 1). Positive comp 1 values can be interpreted as a descriptor of diets dominated by invertebrates, while negative values are indicative of breeding pairs preying mainly on micromammals. Dashed lines represent the confidence intervals of the regression lines.

Dietary parameters were similar for urban and rural burrowing owls. However, we found weak relationships between the proportion of micromammals in the diet and FID (flight initiation distance; positive relationship) and diet diversity and the distance to the nearest breeding pair (nnd; positive relationship), regardless of the habitat where they were found (Table 1). Thus, pairs with larger FID tended to consume more micromammals than pairs formed by bolder (shorter FID) individuals (deviance explained by FID: 1.50%; Fig. 3 and S2). Besides, pairs distant from others showed a more diverse diet than those experiencing higher levels of intraspecific competition (deviance explained by nnd: 5.17%; Fig. 3 and S3). Models fitted separately for each habitat showed that the proportion of micromammals in the diet of urban breeding pairs was related to their behavior, shyer individuals consuming more micromammals than bolder ones. However, the diet of rural pairs was not affected by individual behavior but by intraspecific competition. Pairs located at larger distances to others had more diverse diets than those located closer. Moreover, pairs located in areas with higher conspecific densities showed diets with a higher proportion of micromammals than more isolated pairs (Table 2).

**Table 2:** The first five models obtained to assess the effects of individual behavioural profiles (FID) and intraspecific competition (measured as the distance to the nearest breeding pair, nnd, and the relative position of each breeding pair within the spatial distribution of the entire population, aggregation) on prey richness and diversity, dietary pattern, and degree of specialization of urban and rural breeding pairs of burrowing owls.

URBAN	Model selection				Model averaging			
	*df*	AICc	ΔAICc	w	Variable	Estimate	2.50%	97.50%
Richness								
Null	3	251.74	0.00	0.33	FID	0.80	− 1.13	2.73
FID	4	253.52	1.78	0.14				
Habitat	4	253.75	2.01	0.12				
Year	4	254.14	2.41	0.10				
Nnd	4	254.18	2.44	0.10				
Diversity								
Null	3	73.76	0.00	0.35	FID	0.09	− 0.09	0.28
FID	4	75.17	1.41	0.17				
Year	4	75.90	2.14	0.12				
Habitat	4	75.98	2.23	0.12				
Nnd	4	76.14	2.38	0.11				
Micromammals								
Null	3	29.50	0.00	0.24	FID*	0.09	0.00	0.20
FID	4	29.55	0.05	0.23	Year (2016)	− 0.06	− 0.24	0.11
Habitat	4	31.45	1.95	0.09				
FID + habitat	5	31.65	2.15	0.08				
Year	4	31.86	2.36	0.07				
Psi								
Null	3	− 46.13	0.00	0.33	Year (2016)	− 0.03	− 0.09	0.04
Habitat	4	− 44.29	1.84	0.13				
FID	4	− 43.93	2.20	0.11				
Nnd	4	− 43.83	2.30	0.10				
Year	4	− 43.76	2.37	0.10				

RURAL	Model selection				Model averaging			
	df	AICc	ΔAICc	w	Variable	Estimate	2.50%	97.50%
Richness								
Null	3	170.36	0	0.27	Nnd	1.39	− 0.3	3.08
Nnd	4	170.36	0	0.27	Aggregation	− 1.31	− 3.01	0.39
Aggregation	4	170.69	0.33	0.23				
FID + Aggregation	5	172.76	2.39	0.08				
FID	4	172.98	2.61	0.07				
Diversity								
Nnd	4	31.59	0.00	0.66	Nnd**	0.20	0.06	0.33
FID + Nnd	5	34.56	2.97	0.15				
Aggregation	4	35.81	4.22	0.08				
Null	3	35.92	4.34	0.08				
FID + Aggregation	5	38.43	6.84	0.02				
Micromammals								
Aggregation	4	20.80	0.00	0.45	Aggregation**	0.48	0.06	0.89
Aggregation + Year	5	23.17	2.37	0.14				
Null	3	23.30	2.50	0.13				
FID + Aggregation	5	23.76	2.96	0.10				
Nnd	4	24.99	4.19	0.05				
PSi								
Null	3	− 32.45	0	0.39				
Nnd	4	− 30.18	2.27	0.12				
Year	4	− 29.95	2.51	0.11				
FID	4	− 29.91	2.55	0.11				
Aggregation	4	− 29.76	2.69	0.1				

Models included year as a fixed factor and the identity of the breeding pair as a random term. Richness: number of prey species in the diet; Diversity: diversity of prey species (Shannon index); Micromammals: proportion of micromammals in the diet; Psi: proportional similarity index used to describe the overlap between the diet of a pair and the diet of the entire population (PSi approaches 1 when pairs consume prey in direct proportion to the entire population, decreasing toward 0 in case of specialization). k: number of parameters, AICc: Akaike Information Criterion corrected for small sample sizes, ΔAICc: the difference between the AICc of model *i* and that of the best model (i.e. the model with the lowest AICc), w: Akaike weights. Estimates and 95% confidence intervals (2.5% and 97.5%) were assessed after model averaging.

## Discussion

The burrowing owl has been traditionally considered as a generalist species across its distribution range^47,52–54^. In the present study, invertebrates, mainly coleopterans, were the most abundant prey in its diet, although, in terms of biomass, the largest amount of food was represented by micromammals, in agreement with previous studies conducted in other areas^47,48,52,55^. Other vertebrates and invertebrates appeared in the pellets but in lower abundances, even if for some breeding pairs its contribution to biomass can be important. However, breeding pairs did not use all of these resources randomly, behaving as specialists that exploit a specific subset of the resources utilised by the population. Although the specific prey composition can change between years, possibly as a consequence of changes in prey availability, breeding pairs consistently relied on one or the other type of prey, mainly coleopterans or micromammals. Moreover, despite the spatial continuity between urban and rural habitats, breeding pairs differed in their stable isotope values of C. This stable isotope is an indicator of the primary carbon source, suggesting that owls foraged in the same habitat where they breed. Despite differences in their behavioural profiles (personalities^9^) and the degree of intraspecific competition, which were weakly related to their diet parameters (proportion of micromammals in the diet and diversity, respectively), urban and rural breeding pairs did not differ in their degree of diet specialization or the composition of their diet.

Toscano et al.^31^ have suggested different pathways by which individual personality can drive diet specialization through differences in their prey selection. However, to our knowledge, no study has tested these relationships. Here, we show a weak link between the personality of burrowing owls and their diet, breeding pairs formed by shy individuals relying preferentially on micromammals while bold ones are more prone to prey on coleopterans. This pattern still remains when analysing urban breeding pairs separately, but not for rural ones. Although variability in prey availability among breeding sites cannot be discarded, differences in the energetic balance of individuals with different personalities could explain this pattern. Under the physiology–performance–behaviour–fitness paradigm^56^, bolder individuals, which are also more exploratory^42^, aggressive, and physically active, would gain and expend energy at higher rates than those expressing the opposite suite of behavioural traits^57,58^. Burrowing owls have two main modes of foraging: 'sit-and-wait' or “active hunting”, with energetic costs being greater in the latter than in the former^59^. We can hypothesize that bold individuals may actively search and hunt coleopterans while shy individuals may hunt rodents by ambushing them. However, further experimental research is needed to properly understand this relationship between individual personality and diet.

Intraspecific competition has been proposed as a driver of individual diet specialization because it can cause resource depletion at high densities^60^, a relationship that has not been observed for all species under in situ conditions^34,41^. Although we did not find an effect of intraspecific competition on diet specialization at the population level, prey diversity was lower in breeding pairs located closer to each other. This may be due to the possibility of exploiting a higher range of resources when breeding pairs have no direct competitors in their vicinity, which could reduce the availability of certain prey^61,62^. When we considered urban and rural pairs separately, our results also show that rural breeding pairs in closer proximity to others (shorter distances to conspecifics) had more diverse diets. In addition, rural pairs in areas with a high density of conspecifics had diets with a higher proportion of micromammals than those that were more isolated. Prey abundance is a limiting factor affecting bird breeding density^63^ and, although we lack estimates of prey availability, the positive relationship between the proportion of micromammals in the diet and the aggregation of individuals may also indicate that high densities of micromammals allow for a higher density of breeding pairs. However, this relationship should be explored in more detail to understand its real meaning, as it could also be due to differences in the aggregation of pairs with different behaviours. In fact, breeding pairs composed of bolder individuals (i.e., lower FID) show a higher aggregation than those composed of more shy individuals (Fig. S4). Although we found no relationship between the behaviour of these individuals and their diet profiles, we cannot rule out that the pattern found could result from a combined effect of food availability and individual behaviour.

Identification of prey remains and pellets, despite known taxonomic biases towards prey with hard structures, has the advantage of being non-invasive, allowing the possibility of resampling individuals over years with minimal disturbance. However, diet estimations using pellets and remains has been largely criticized because they may reflect only a snapshot of a consumer’s diet^64^. Here, we complementary used stable isotope analysis, in particular nitrogen (δ^15^N), which allowed us to confirm that the diet of owls sampled is representative of the dietary pattern of breeding pairs (micromammals or coleopterans) over longer temporal windows (weeks). Although we have not analysed the stable isotope values of the prey, it is well known that low nitrogen values are related to prey placed at low trophic positions (herbivores), such as rodents, while the consumption of prey placed at higher trophic positions (predators and detritivores), such as many beetles, have higher nitrogen values.

In conclusion, our results are in line with previous research showing how generalist species or populations can be ultimately composed of individuals (or breeding pairs in our case) with different degrees of specialization. This specialization, which does not seem to be related to the occupation of human-modified habitats but to individual behaviour (measured through fear of humans^9,42^) and intraspecific competition, can have long-lasting effects on the demographic parameters of individuals, considering the different nutritional contributions of prey (coleopterans vs micromammals^65,66^). In this sense, more research is needed to understand the potential effects of trophic specialization and differences in resource use in the physiological state of individuals and on the development of behaviors^66^. These studies should also consider the role of spatio-temporal variability in resource distribution on individual specialization^17^, something we cannot rule out given the absence of data on the availability of the different prey used by owls in our study area. Therefore, we encourage further work in this line of research, compiling detailed information to properly unravel the role of conspecifics, individual behaviour and resource availability in the trophic specialization of species.

## Materials and methods

### Study species and area

The burrowing owl inhabits North and South American grasslands. The species has been described as a generalist predator of invertebrates (mainly insects and arachnids) and vertebrates (preying on micromammals, reptiles and birds^52–55^) throughout its range, from Canada in the north to the Patagonian region in the south^55,67^. Although in its northern range the species has experienced a significant decline during the last decades, it is still abundant in its southern range^68^. In our study area, which includes the city of Bahía Blanca (Buenos Aires, Argentina) and its surrounding rural areas (ca. 5400 km^2^), rural owls breed in natural grasslands and pastures dedicated to cattle raising where human presence is rare and mostly restricted to some scarce roads and scattered farms. Urban owls, conversely, excavate their nests in private gardens, public parks, unbuilt spaces among houses, roundabouts, and large avenues, in continuous contact with people and traffic. Urban and rural habitats are continuously distributed, without clear habitat interface between them, as urbanized areas are immediately surrounded by rural ones (see 33 for further details).

### Fieldwork procedures

During the breeding season of the burrowing owls (October–February), we survey the study area -almost daily- from 2006 to 2020 to locate breeding pairs and active nests. Owl nests are easily located since breeders show diurnal activity and usually perch in the entrance of their burrows or on nearby small bushes and fences^33^. We estimated the diet of burrowing owls by collecting pellets and prey remains in urban and rural entrance nests during the chick-rearing periods (December–February) in two consecutive years differing in annual precipitation records (2015: 769 mm and 2016: 653 mm). Analyses of pellets and food remains are the most common methods to study bird diet, despite their limitations and biases, linked to prey sizes or digestibility^69,70^. However, colleting both (pellets and prey remains) may reduce these biases^71^. Collections were made twice in nests occupied by individuals that were previously marked with colour-numbered plastic rings to have information across years for the same breeding pairs (see 9 for details on the long-term burrowing owls monitoring program in the study area). Blood samples were taken during the chick marking process from both chicks and adults recaptured at that time. However, due to the impossibility of differentiating prey obtained by males or females, our sampling units were the breeding pair. Pellets and prey remain were individually packaged, dried and preserved on aluminum foil in a freezer (− 4 °C) to reduce the risk that bacteria or fungi could deteriorate them until laboratory analysis.

Diet estimations using pellets and remains often reflect only a snapshot of a consumer’s diet, so we indirectly characterized the diet of burrowing owls using stable isotope analyses, assuming that the isotopic signatures of different dietary sources are predictably reflected in consumer tissues^64^. We focused on nitrogen (δ^15^N) and carbon (δ^13^C) stable isotopes, which have been reportedly highlight as a useful tool in avian ecology for providing an integrated view of resource consumption, identifying feeding strategies and trophic levels of species over long periods^64,72^. Hence, we studied the diet composition of the burrowing owl combining two approaches: a non-invasive, direct method (pellets and prey remains from nests) and an indirect one by stable isotopes. This combination has been pointed out as suitable to minimize bias in prey consumption determination for several avian species (e.g. European Roller^73^; Bonelli´s Eagle^74^; Lesser kestrel^75^). We analysed the stable isotope values in blood samples, which typically inform diet composition over a time frame of 2–4 weeks^76^. Blood was obtained from the brachial vein of individuals captured (0.2 ml) with bow nets and ribbon carpets at each nest (adults and/or chicks) and preserved in absolute ethanol at 4 °C until processing in the laboratory.

We used the location of all breeding pairs to calculate the distance of each pair to its nearest neighbour (nnd) and relative position within the spatial distribution of all breeding pairs (aggregation; calculated as *Si* = *Σ exp* (− *dij*) (with *i* ≠ *j*), where *dij* was the linear distance between nest *i* and *j*) as indicators of intraspecific food competition at small and large spatial scales, respectively. To assess potential trophic niche differences of breeding pairs associated with the behavioural profile of individuals, we measured the flight initiation distances (FIDs) of breeders during the chick-rearing period. FID has been previously related to individual personalities, correlating with exploration and antipredator response^42^. Briefly, we measured FID by walking towards focal individuals, which were perched on fences or other similar structures or close to the ground near their nests, following a direct trajectory, with no obstacles blocking the bird and the observer and at a constant speed of 0.5 m/s. Distances at which birds fledged were measured with a laser telemeter incorporated into 10 × 42 binoculars (Leica Geovid, range: 10–1300 m) or by counting steps for distances less than 10 m^26^. FIDs were measured during the day, when owls were active and easily located from a distance, given the bare ground and short vegetation surrounding their nests. We used as an indication of a bird’s behaviour one FID value per individual or the mean when more than one value was available because of its high within-individual repeatability^9,26^. As breeders were not randomly mated for fear of humans (Spearman correlation = 0.78, *p* < 0.0001, *n* = 628; see also^9,26^), we used the average FIDs of both members of a breeding pair (n = 17) or the FID of the male (n = 5) or female (n = 14) as indicative of each pair behaviour (information was not available for the remaining 4 breeding pairs; see 28 for a similar approach). Individuals were sexed based on plumage characteristics or, when necessary, by molecular procedures^44^.

### Laboratory procedures

Pellets and prey remains were weighed (in g) to obtain an estimate of sampling effort and to standardize diet data among breeding pairs. Each pellet was analysed as an independent sample, as well as each prey remain, to identify and classify the prey items to the finest taxonomic level^47,53^ using a dissecting microscope and taxonomical keys and field guides for determination. Arthropods were classified using their chitinous remains and hard body parts, as elytra, legs, mandibles, and heads^55^, which were also used to estimate their abundances (minimum number of individuals). Vertebrates, mainly micromammals, were identified using cranial remains and mandibles^55^, and their abundances were estimated by counting the total number of skulls or the maximum number of left and right mandibles^53^. Reptiles, amphibians, and birds were determined by feathers, skulls, or other well-preserved structures. The biomass of each prey item was estimated using its average fresh weight (in g) obtained with the help of specialists and various bibliographic sources^77–81^.

Blood samples were lyophilized for 24 h with a Telstar Cryodos-50 freeze-dryer and then manually ground to powder. Samples, ranging from 0.300 to 350 mg, were placed in tin capsules. All samples were oxidized in a Flash EA1112 Elemental Analyzer and TC-EA pyrolyzer coupled to a Delta C Finnigan MAT mass spectrometer through a Conflo III interface (ThermoFinnigan), where δ^15^N and δ^13^C signatures were determined using Isotopic ratio mass spectrometry at the Laboratorio Isótopos Estables, Estación Biológica de Doñana, CSIC, Spain. [(R_sample_/R_standard_) − 1]*1000, where *X* (‰) is ^15^N and ^13^C, and *R* are the corresponding ^15^N/^14^N and ^13^C/^12^C ratios, related to standard values (*R*_standard_: ^15^N: atmospheric nitrogen (AIR); ^13^C: Vienna Pee Dee Belemnite, VPDB). International standards (IAEA CH_7_ and IAEA CH_6_ for C, IAEA N_1_ and IAEA N_2_ for N, USGS 34, USGS 40, and acetanilide for both C and N) were run every 12 samples to calibrate the system. Replicated assays of standard materials indicated measurement errors of ± 0.2 and ± 0.1‰ for nitrogen and carbon, respectively.

### Statistical analyses

We estimated the richness and diversity (Shannon index^82^) of prey taxa in the diet of each breeding pair during both chick rearing periods. We then identified their dietary patterns using Multiple Factor Analysis (MFA). MFA is an extension of principal component analysis (PCA) that handles multiple data tables measuring sets of variables repeatedly collected on the same individuals^83^. MFA first calculates a PCA of each data table (in our case, one for each year) and "normalises" them by dividing all their elements by the first singular value obtained from their PCA. Then, these tables are aggregated into a large data table that is analysed by a (non-normalised) PCA that provides a set of factor scores for the observations and loadings for the variables. Qualitative variables included in MFA were the proportion of the diet biomass of each breeding pair corresponding to micromammals and coleopterans (the main prey items identify in the study area), the proportion of the diet biomass corresponding to other vertebrates (birds, amphibians, and reptiles), and the proportion of the diet biomass corresponding to other invertebrates such as arachnids, orthopterans, or hymenopterans. All variables were scaled prior to analysis. Eigenvalues > 1 were used to assess the number of factors to extract. We used linear mixed models to relate δ^15^N and δ^13^C values (dependent variables) to the dietary pattern (estimated through factors extracted from MFA and the original variable or variables that most correlates with them) of the breeding pairs, including the year and habitat type (urban or rural) as fixed factors in models and the identity of the breeding pair as a random term.

Variation in the diet between pairs and their degree of specialization was assessed by analysing the realized trophic niche using the R package “RInSp”^84^. For urban and rural breeding pairs, we estimated their Total Niche Width (TNW) using the equations proposed by Roughgarden^85^ modified for discrete data, using the Shannon index as a proxy for variance. The TNW was decomposed into two components: Between Pair Component (BPC) and Within Pair Component (WPC). The WPC/TNW ratio is a measure of between pair diet variation, values close to 1 indicate low between-pair diet variation, while values close to 0 indicate decreased between-pair overlap and increased specialization. The statistical significance of WPC/TNW was assessed by Monte Carlo simulations, running 999 simulated populations in which the same number of pairs present in the real population randomly choose dietary items from the population resource distribution. The WPC/TNW ratio is then recalculated for each of these simulated populations composed of generalist pairs (null models). A non-parametric *p* value was obtained by considering the proportion of simulated populations that had higher values than those observed in the real one. Complementarily, we used the proportional similarity index (Psi) to describe the overlap between the diet of a pair and the diet of the entire population^86^. As before, PSi approaches 1 when pairs consume prey in direct proportion to the entire population, decreasing toward 0 in case of specialization. The IS index, calculated as the average PSi of pairs, represents a general measure of specialization at the population level. The statistical significance of this index was calculated following the same simulation approach as for the WPC/TNW ratio. One advantage of these indexes is that, instead of measuring niche breadth by comparing the species’ (or population’s) resource frequency distribution within available resources, they use the entire diet of the species or population to define resource availability^86^. This approach is especially useful for species that exploit a wide variety of vertebrate and invertebrate prey, as is the case in our study model, which would require a variety of standardized sampling techniques across taxa to obtain reliable and comparable estimates of prey availability. Thus, the resource use of pairs is compared to their population rather than to the environment.

We estimated the repeatability of the different diet parameters between years using the null models (i.e., models without including explanatory variables) in the rptR library^87^. We used linear mixed models to assess differences in prey richness and diversity, dietary pattern, and degree of specialization (dependent variables) between urban and rural pairs (habitat, fixed factor), considering their behavioural profiles (FID, continuous variable) and level of intraspecific competition (nnd or aggregation, continuous variables). As both measures of intraspecific competition were correlated (Pearson correlation test: r = − 0.45, t = − 4.13, *p* = 0.0001), we used them alternatively in models. The year was included in models as a fixed effect and the identity of the breeding pairs as a random term. As rural pairs were composed of shyer individuals (FID: mean = 48.21 m, sd = 28.09 m) and were subject to higher intraspecific competition (nnd: mean = 0.30 km, sd = 0.37 km; aggregation: mean = 29.27, sd = 13.08) than their urban counterparts (FID: mean = 21.65 m, sd = 15.36 m, χ^2^ = 28.50, *df* = 1, *p* < 0.0001; nnd: mean = 0.11 km, sd = 0.06 km, χ^2^ = 16.45, *df* = 1, *p* < 0.0001; aggregation: mean = 20.38, sd = 13.96, χ^2^ = 6.70, *df* = 1, *p* = 0.0086; Fig. S1), we fit the same models separately for urban and rural breeding pairs. All the statistical analyses were performed using the glmmTMB package^88^ in R 4.1.0^89^. Model selection was performed using the Akaike Information Criterion corrected for small sample sizes, AICc^90^. Within each set of models (which includes the null model but not models that did not converge), we calculated the ΔAICc (as the difference between the AICc of model *i* and that of the best model) and the Akaike weight (w) of each model. Models within 2 AICc units of the best one were considered as alternatives and, when needed, used to perform model averaging (package MuMIn^91^). We considered that a given effect received no, weak, or strong support when the 95% confidence interval (CI) strongly overlapped zero, barely overlapped zero, or did not overlap zero, respectively. The fit of the final models was tested using the package DHARMa^92^, which employed a simulation-based approach to create standardized residuals (values between 0 and 1) for fitted (generalized) linear (mixed) models and test the significance of the dispersion parameter, zero-inflation, and goodness-of-fit of the model (H_0_: fitted model suits well for the data).

### Ethics statements

Fieldwork and procedures were conducted under permits from the Argentinean wildlife agency (22500-4102/09), and the owners of private properties, in accordance with the approved guidelines of the Consejo Superior de Investigaciones Científicas CSIC (CEBA-EBD-11-28). This study was approved by the Ethics Committee of the Consejo Superior de Investigaciones Científicas CSIC, and complies with the ARRIVE guidelines.

### Ethics approval

Fieldwork and procedures were conducted under permits from the Argentinean wildlife agency (22500-4102/09), and the owners of private properties, under the approved guidelines of the Ethics Committee of CSIC (CEBA-EBD-11-28).

## Supplementary Information

Supplementary Information.

## Data Availability

The datasets used and/or analysed during the current study are available from the corresponding author on reasonable request.
