# Supplementary material for: Intraspecific competition and individual behaviour but not urbanization affect the dietary patterns of a generalist avian predator
Source: Sci Rep. 2023 Jun 24;13:10255. doi: 10.1038/s41598-023-37026-y (PMC10290650; doi:10.1038/s41598-023-37026-y)
Supplement: Supplementary file 1 — Supplementary Information. [file 41598_2023_37026_MOESM1_ESM.docx]

# **SUPPLEMENTARY MATERIAL**

**Intraspecific competition and individual behaviour but not urbanization affect the dietary patterns of a generalist avian predator**

Pedro Romero–Vidal^1,2,*^, Álvaro Luna^3^, Lola Fernández–Gómez^4^, Joan Navarro^5^, Antonio Palma^2^, José L. Tella^2^, Martina Carrete^1^

**Fig. S1.** Fear of humans (measured as fid, flight initiation distance, in m), and distance of each pair to its nearest conspecific neighbour (nnd) and the relative position within the spatial distribution of all breeding pairs of urban and rural burrowing owl breeding pairs. Dots represent the mean values and bars represent standard errors. For each variable, the Kruskal-Wallis test result and its significance are included.


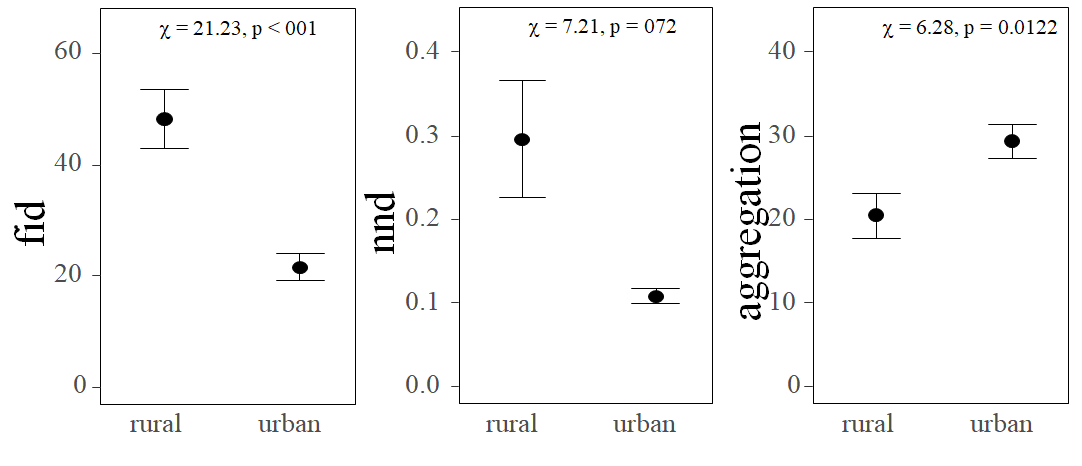


**Fig. S2.** qq–plot and standard residuals plots for the model obtained to describe the relationship between FID and PC1. No significant problems were detected.


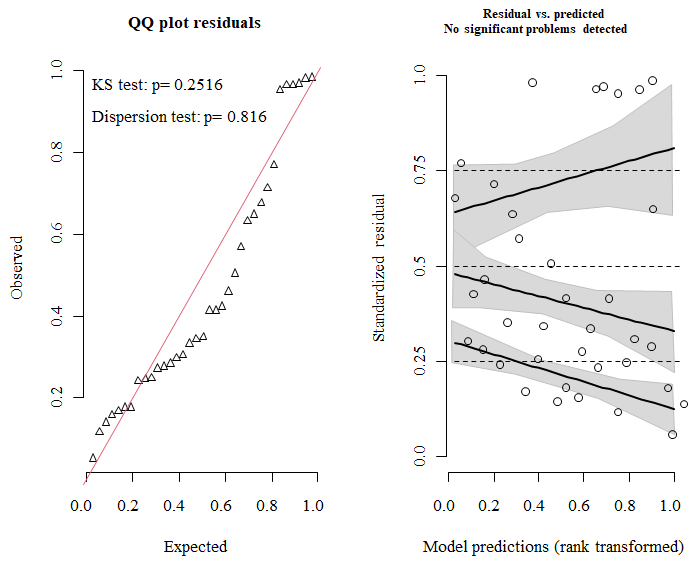


**Fig. S3.** qq–plot and standard residuals plots for the model obtained to describe the relationship between diet diversity and intraspecific competition (measured through the distance to the nearest breeding pair, nnd). No significant problems were detected.


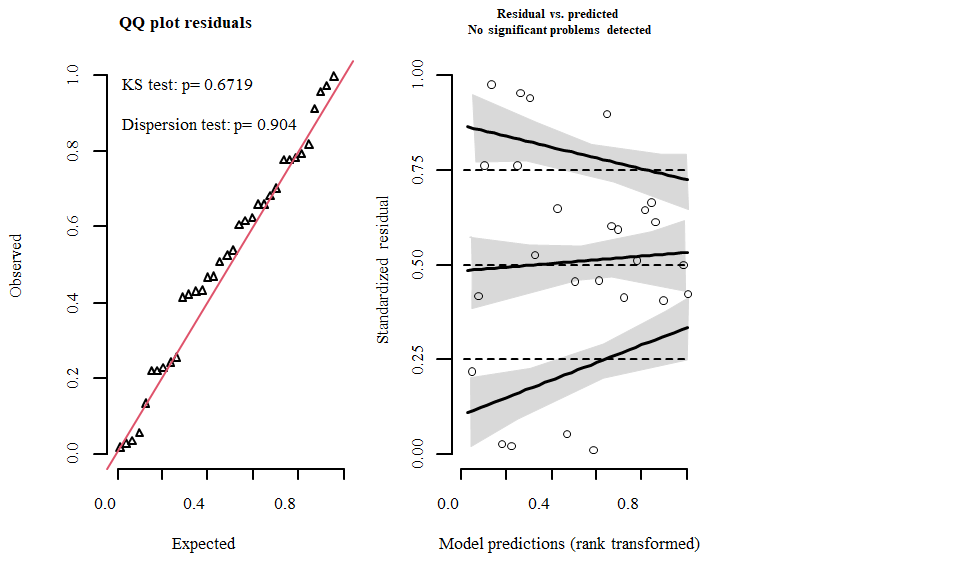


**Fig. S4.** Relationship between individual behavioural profiles (FID) and intraspecific competition (measured as the relative position of each breeding pair within the spatial distribution of the entire population, aggregation) among rural burrowing owls.


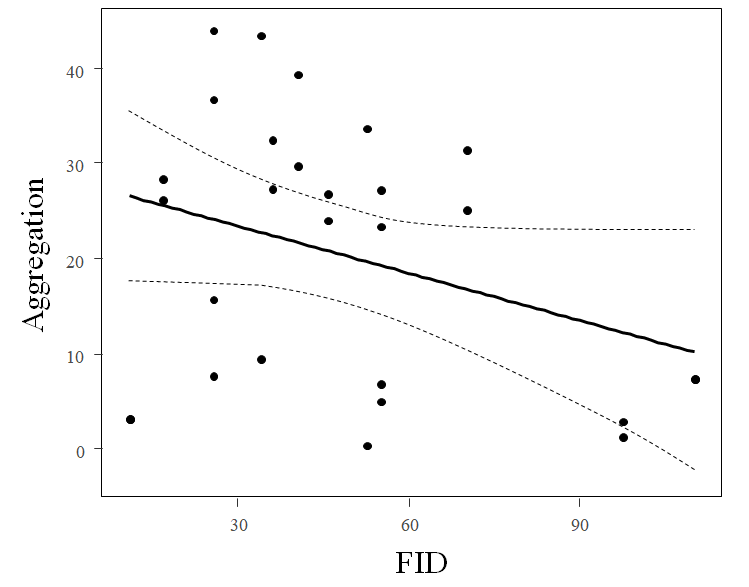


**Table S1.** Prey consumption, estimated as the total number of individuals identified for a specific group over the total number of individuals consumed in urban and rural habitats, and by the total population.

| **Taxa** | **Total pop** | | **Rural** | | **Urban** | |
| --- | --- | --- | --- | --- | --- | --- |
|  | **Number** | **Percentage** | **Number** | **Percentage** | **Number** | **Percentage** |
| Insecta |  |  |  |  |  |  |
| Coleoptera | 3875 | 65.79% | 1655 | 65.57% | 2220 | 65.95% |
| Orthoptera | 121 | 2.05% | 53 | 2.10% | 68 | 2.02% |
| Himenoptera | 54 | 0.92% | 16 | 0.63% | 38 | 1.13% |
| Mantoidea | 1 | 0.02% | 0 | 0.00% | 1 | 0.03% |
| Hemiptera | 2 | 0.03% | 1 | 0.04% | 1 | 0.03% |
| Arachnida | 694 | 11.78% | 248 | 9.83% | 446 | 13.25% |
| Gastropoda | 19 | 0.32% | 13 | 0.52% | 6 | 0.18% |
| Malacostraca  Isopoda | 294 | 4.99% | 120 | 4.75% | 174 | 5.17% |
| Amphibia | 52 | 0.88% | 15 | 0.59% | 37 | 1.10% |
| Reptilia | 16 | 0.27% | 8 | 0.32% | 8 | 0.24% |
| Aves | 40 | 0.68% | 20 | 0.79% | 20 | 0.59% |
| Mammalia  Rodentia | 722 | 12.26% | 375 | 14.86% | 347 | 10.31% |

**Table S2.** Results of the Multiple Factor Analysis (MFA), including the first 5 components (comp) and the scores obtained for each independent variable for each factor.

|  | comp 1 | comp 2 | comp 3 | comp 4 | comp 5 |
| --- | --- | --- | --- | --- | --- |
| eigenvalue | 1.43 | 0.69 | 0.61 | 0.52 | 0.34 |
| %variance | 38.83 | 18.72 | 16.43 | 14.21 | 9.34 |
| 2015 |  |  |  |  |  |
| Micromammals | -0.85 | -0.27 | 0.31 | -0.31 | -0.04 |
| Coleopterans | 0.83 | 0.36 | -0.31 | -0.12 | -0.03 |
| Other invertebrates | 0.28 | -0.37 | -0.36 | 0.62 | 0.51 |
| Other vertebrates | 0.44 | 0.53 | 0.2 | 0.46 | -0.47 |
| 2016 |  |  |  |  |  |
| Micromammals | -0.82 | 0.36 | -0.42 | 0.17 | -0.04 |
| Coleopterans | 0.71 | -0.04 | -0.26 | -0.61 | 0.08 |
| Other invertebrates | 0.46 | -0.75 | 0.28 | 0.2 | -0.29 |
| Other vertebrates | 0.23 | 0.41 | 0.77 | 0.03 | 0.43 |

**Table S3.** Repeatability (and its 95% confidence interval) in the proportion of the biomass of the diet of burrowing owl pairs corresponding to Coleopterans, other invertebrates (spiders, orthopterans, etc.) and other vertebrates (birds, reptiles and amphibians), calculated using the null model (i.e. models without explanatory variables).

| Diet parameter | Repeatability |
| --- | --- |
| Coleopterans | 0.61 (0.34, 0.77) |
| Other invertebrates | 0.05 (0, 0.37) |
| Other vertebrates | 0.09 (0, 0.4) |
